# Exploring the Association Between Physical Fitness (High Intensity and Low Intensity) and the Incidence of Temporomandibular Disorders: A Comparative Study

**DOI:** 10.7759/cureus.66047

**Published:** 2024-08-02

**Authors:** Abhishek Verma, Nishath Sayed Abdul, Anindita Bhagawati, Tribeni Saikia, Amritaksha Bhattacharya, Pawan Rajendra Joshi, Sahana Shivakumar, Shivakumar G C

**Affiliations:** 1 Public Health Dentistry, Sardar Patel Post Graduate Institute of Dental & Medical Sciences, Lucknow, IND; 2 Oral Pathology and Oral Diagnostic Sciences, College of Medicine and Dentistry, Riyadh Elm University, Riyadh, SAU; 3 Oral Surgery, Government Dental College, Dibrugarh, Dibrugarh, IND; 4 Orthodontics and Dentofacial Orthopedics, Government Dental College, Dibrugarh, Dibrugarh, IND; 5 Oral Pathology and Microbiology, Government Dental College, Dibrugarh, Dibrugarh, IND; 6 Pediatric and Preventive Dentistry, Vidarbha Youth Welfare Society (VYWS) Dental College and Hospital, Amravati, IND; 7 Public Health Dentistry, People's College of Dental Sciences & Research Centre, Bhopal, IND; 8 Oral Medicine and Radiology, People's College of Dental Sciences & Research Centre, Bhopal, IND

**Keywords:** pain, temporomandibular disorders, low-intensity training, high-intensity training, fonseca anamnestic index

## Abstract

Background: Temporomandibular joint disorders affect the temporomandibular joint (TMJ), masticatory muscles, and associated structures. Symptoms include TMJ pain, limited jaw movement, muscle tenderness, and referred pain. Physical activity can alleviate musculoskeletal pain. This study explored the link between physical fitness (high and low intensity) and temporomandibular disorder (TMD) incidence.

Methodology: Sixty patients were divided into three groups in this comparative study. Group I underwent 30 minutes of high-intensity aerobic training. Group II had 30 minutes of low-intensity yoga sessions weekly. Group III received health education. TMD was diagnosed using the Fonseca Anamnestic Index (FAI). Pain intensity was measured using the Visual Analogue Scale (VAS) and the Pain Self-Efficacy Questionnaire (PSEQ).

Results: Of the participants, 38.1% were males and 61.9% were females. TMD severity was mild (25.0%), moderate (55.0%), and severe (20.0%). High-intensity training groups had higher TMD symptom severity than low-intensity groups (p = 0.001). VAS scores increased in group I and decreased in group II (significant). PSEQ scores decreased in group I and increased significantly in group II. Group III showed no significant differences in PSEQ scores.

Conclusion: High-intensity training resulted in moderate TMD symptoms. Low-intensity training was beneficial for TMD pain. The study recommends combining low-intensity physical workouts with medications to alleviate TMD.

## Introduction

Temporomandibular joint disorders are defined as a class of diseases that mainly impact the masticatory muscles, the temporomandibular joint, and the tissues that are related to them. Tenderness in the masticatory muscles, referred pain in the head, neck, ear, and dental areas, and pain and clicking sounds in the temporomandibular joint with restricted jaw mobility are the most typical indications and symptoms of temporomandibular disorder (TMD) [1,2]. Approximately 37% of adult Indians suffer from temporomandibular joint dysfunction. According to research, between 15-50% and 30-90% of people, respectively, have at least one symptom and one clinical indication of TMD [3,4]. These have a complex etiology. A complex interplay is played by metabolic processes, genetic variables, growth and developmental factors, psychological stress, and parafunctional behaviors [5]. Using the Fonseca Anamnestic Index (FAI), the signs, symptoms, and severity of the condition are used to diagnose TMDs. These days, a person's lifestyle and health-related behaviors are also very important in determining the cause of TMD and chronic pain [6]. In TMDs, persistent discomfort and jaw movements are also substantially correlated with alcohol use, snuff use, tobacco smoking, and obesity [7,8]. According to Wanman, individuals with TMDs are less physically capable of carrying out a variety of tasks involving the muscles of the jaw and shoulder [9].

Through the release of endocannabinoids, or beta-endorphins, physical activity reduces sensitivity to pain stimuli [10]. Physical activity of any form has been shown to help with musculoskeletal pain by influencing the autonomic and cognitive elements of pain. Additionally, it has been demonstrated that exercise can affect the symptoms and indicators of a number of pain-related conditions [2,8], as demonstrated by Kraus et al., who showed that patients with osteoarthritis can have pain relief and improved joint functioning with physical fitness training [11]. On the other hand, certain research findings indicate that people with fibromyalgia and lower back issues who engage in high-intensity physical exercise experience intense discomfort [5]. While the mechanism underlying these reactions remains poorly understood, some researchers believe that intense physical exercise raises inflammatory mediators and consequently increases pain, while others speculate that moderate-to-intense physical activity is linked to decreased levels of C-reactive protein and inflammatory responses [12].

In the context of managing chronic pain, TMD, which is extremely common and has a substantial negative impact on quality of life, has been connected to physical fitness. The current study compared high-intensity and low-intensity training sessions to examine the effect of physical training on the incidence of TMD and address this relationship.

## Materials and methods

Study design and sampling

A comparative analysis was conducted to investigate the impact of high-intensity and low-intensity training on the incidence and severity of TMDs. After reviewing the study, the People's College of Dental Sciences and Research Centre, Bhopal, Institutional Ethical Committee granted approval (Ethical clearance number: EC244224). After outlining the purpose, goals, and methodology of the study, each participant provided written informed consent.

Sixty patients were recruited for the study. Power analysis was done to establish the adequacy of a sample size of 60 for investigating the association between physical training and the occurrence of TMDs. Parameters employed for power analysis were a significance level (α) of 0.05, a power (1-β) of 0.80, and a sample size of 60. The power analysis demonstrated an effect size (Cohen's d) of approximately 0.52, which is a medium effect size. This indicates that, with a sample size of 60, the study has an 80% chance of detecting a medium effect size at the 5% significance level.

Participant eligibility criteria

Adult patients aged more than 18 years who were diagnosed with TMDs were included. The diagnosis of TMD cases was performed as per the FAI [13]. The FAI is a self-reported questionnaire developed by Dr. Djalma Fonseca to screen TMD patients in epidemiological surveys and clinical settings. It is a widely utilized diagnostic tool designed to assess the signs and symptoms associated with TMDs. Patients suffering from lower back pain, rheumatoid arthritis, spondylitis, any systemic musculoskeletal and endocrine disorders, liver problems, history of trauma within six months, sleep disturbances, psychiatric disorders or distress, and being on medication for any of these underlying conditions were excluded. The examiners were blinded to all evaluations.

Intervention

A baseline assessment of patients was done using the FAI index. Following this, group I received high-intensity training, group II received low-intensity training, and group III received no physical training but only a health education session (control group).

Group I received a 30-minute session of high-intensity training weekly for four weeks in the form of aerobics and weight lifting. The intervention started with a five-minute warm-up session followed by 25 minutes of high-intensity training and a two-minute recovery period.

Group II received a 30-minute session of low-intensity training weekly for four weeks in the form of yoga, which was performed by the same instructor. Yoga sessions consisted of warm-ups, breathing exercises, and leisure asanas. In the warm-up, stretching of muscles was performed, focusing on upper extremity and neck movements, followed by various asanas like varaksana, padahastasana, and sasankasana [14]. Lastly, some leisure activities were performed for muscle relaxation.

Group III received only health education, and no form of physical training was advised. This ensured that the control group received attention and information similar to the intervention groups, facilitating the control for the placebo effect and ensuring that any differences observed in the outcomes were solely attributed to the specific physical training interventions.

Data collection

Baseline information was collected from all three groups through the Visual Analogue Scale (VAS) and Pain Self-Efficacy Questionnaire (PSEQ). Pain assessment was done again after four weeks of intervention and follow-up. The Cronbach's alpha (reliability, validity, and internal consistency) value of this questionnaire was reported to be 0.92 [15]. VAS was used to check the severity of pain before and after four weeks of intervention [16].

Statistical analysis

Data were entered into Excel spreadsheets (Microsoft Corporation, Redmond, WA), and SPSS version 25.0 (IBM Corp., Armonk, NY) was used for statistical analysis. The chi-square test was used to examine the relationship between the groups for the FAI and VAS scale, while one-way ANOVA was used to evaluate the PSEQ mean value. The post hoc analysis was used to verify the intergroup comparison. A p-value of less than 0.05 was deemed significant for every analysis.

## Results

In this study, a total of 60 patients were diagnosed with TMDs with ages ranging from 18 to 40 years, with a mean age of 24.83 ± 5.334 years. Out of which, 23 (38.3%) were males and 37 (61.7%) were females. The TMD severity assessment showed that 15 (25.0%) patients had mild TMD, 33 (55.0%) had moderate TMD, and 12 (20.0%) had severe TMD. FAI showed no significant difference between the three groups before intervention. After the intervention, it was observed that the severity of TMD symptoms increased among high-intensity training groups, whereas it was reduced in low-intensity training groups, significant at p = 0.001, as shown in Table 1.

**Table 1:** Severity of temporomandibular disorders among groups before and after intervention. FAI: Fonseca Anamnestic Index; TMJ: temporomandibular joint.

FAI questions	Choices	Before intervention			P-value	After intervention			P-value
		Group I	Group II	Group III		Group I (high-intensity training group)	Group II (low-intensity training group)	Group III (control group)	
Q1. Do you have difficulty opening your mouth wide?	Yes	14 (70%)	16 (80%)	13 (65%)	0.563	18 (90%)	8 (40%)	9 (45%)	0.010
	No	6 (30%)	4 (20%)	7 (35%)		2 (20%)	12 (60%)	11 (55%)	
Q2. Do you have difficulty moving your jaw side?	Yes	14 (70%)	16 (80%)	13 (65%)	0.563	18 (90%)	8 (40%)	9 (45%)	0.010
	No	6 (30%)	4 (20%)	7 (35%)		2 (20%)	12 (60%)	11 (55%)	
Q3. Do you feel fatigue or muscle pain when chewing?	Yes	12 (60%)	12 (60%)	14 (70%)	0.750	14 (70%)	3 (15%)	10 (50%)	0.05
	No	8 (40%)	8 (40%)	6 (30%)		6 (30%)	17 (85%)	10 (50%)	
Q4. Do you have frequent headaches?	Yes	12 (60%)	14 (70%)	12 (60%)	0.750	16 (80%)	8 (40%)	10 (50%)	0.126
	No	8 (40%)	6 (30%)	8 (40%)		4 (20%)	12 (60%)	10 (50%)	
Q5. Do you have neck pain or wryneck?	Yes	12 (60%)	14 (70%)	12 (60%)	0.47	16 (80%)	9 (45%)	9 (45%)	0.036
	No	8 (40%)	6 (30%)	8 (40%)		4 (20%)	11 (55%)	11 (55%)	
Q6. Do you have earaches or pain in the TMJ?	Yes	13 (65%)	13 (65%)	14 (70%)	0.928	16 (80%)	9 (45%)	9 (45%)	0.036
	No	7 (35%)	7 (35%)	6 (30%)		4 (20%)	11 (55%)	11 (55%)	
Q7. Have you noticed any clicking in your TMJ while chewing or opening your mouth?	Yes	12 (60%)	9 (45%)	13 (65%)	0.414	14 (70%)	8 (40%)	12 (60%)	0.150
	No	8 (40%)	11 (55%)	7 (35%)		6 (30%)	12 (60%)	8 (40%)	
Q8. Have you noticed if you have a habit of clenching or grinding your teeth?	Yes	13 (65%)	11 (55%)	13 (65%)	0.754	13 (65%)	9 (45%)	15 (75%)	0.139
	No	7 (35%)	9 (45%)	7 (35%)		7 (35%)	11 (55%)	5 (25%)	
Q9. Do you feel your teeth do not articulate well?	Yes	13 (65%)	15 (75%)	9 (45%)	0.139	14 (70%)	15 (75%)	9 (45%)	0.108
	No	7 (35%)	5 (25%)	11 (55%)		6 (30%)	5 (25%)	11 (55%)	
Q10. Do you consider yourself a tense (nervous) person?	Yes	14 (70%)	13 (65%)	11 (55%)	0.605	11 (55%)	10 (50%)	13 (65%)	0.622
	No	6 (30%)	7 (35%)	9 (45%)		9 (45%)	10 (50%)	7 (35%)	

In the current study, 23 (38.3%) patients reported moderate pain, while 37 (61.6%) patients experienced severe pain before intervention. Statistically, no significant difference was observed in pain intensity between groups (p = 0.493) before intervention. After intervention, pain intensity in group I individuals remained the same (60% versus 60%). In group II, none of the patients had severe pain (70% versus 0%). This difference in pain severity after intervention was significant at p = 0.001, as shown in Table 2.

**Table 2:** Interpretation of pain among groups before and after intervention.

Groups	Before intervention					After intervention (8 weeks)				
	No pain, n (%)	Mild pain, n (%)	Moderate pain, n (%)	Severe pain, n (%)	P-value	No pain, n (%)	Mild pain, n (%)	Moderate pain, n (%)	Severe pain, n (%)	P-value
Group I (high-intensity training)	0 (0%)	0 (0%)	8 (40%)	12 (60%)	0.803	0 (0%)	6 (30%)	2 (10%)	12 (60%)	0.001
Group II (low-intensity training)	0 (0%)	0 (0%)	6 (30%)	14 (70%)		14 (70%)	4 (20%)	2 (10%)	0 (0%)	
Group III (control group)	0 (0%)	0 (0%)	9 (45%)	11 (55%)		0 (0%)	3 (15%)	9 (45%)	8 (40%)	
Total	0 (0%)	0 (0%)	23 (38.3%)	37 (61.6%)		14 (23.3%)	13 (21.6%)	13 (21.6%)	20 (33.3%)	

When mean VAS scores were compared, group I individuals showed a significant increase from 2.22 ± 0.712 to 2.39 ± 0.584 (p = 0.001). In group II, the VAS mean score was significantly reduced from 2.08 ± 0.700 to 1.08 ± 1.109 (p = 0.001). No significant change in the mean score of group III patients was observed, as shown in Table 3.

**Table 3:** Comparison of mean scores on the VAS scale before and after intervention. VAS: Visual Analogue Scale.

Groups	Before intervention	After intervention (4 weeks)	P-value
Group I (high-intensity training)	2.30 ± 0.923	2.60 ± 0.503	0.055
Group II (low-intensity training)	1.75 ± 0.444	0.40 ± 0.681	0.001
Group III (control group)	2.55 ± 0.510	2.35 ± 0671	0.214

Similarly, PSEQ evaluation showed an increase in scores from 18.17 ± 4.426 to 44.86 ± 5.522 in group II, while it decreased from 17.82 ± 4.438 to 12.08 ± 1.625 in group I, which was significant, as shown in Table 4.

**Table 4:** Mean value of the PSEQ before and after interpretation among groups. PSEQ: Pain Self-Efficacy Questionnaire.

Groups	Before intervention	P-value	After intervention	P-value
	Mean value of PSEQ		Mean value of PSEQ	
Group I (high-intensity training)	17.15 ± 4.283	0.410	12.10 ± 1.683	0.001
Group II (low-intensity training)	18.45 ± 4.662		43.05 ± 5.286	
Group III (control group)	19.00 ± 4.449		23.95 ± 1.504	

On post hoc comparison, the mean differences of PSEQ in groups II and III concerning group I were 1.317 ± 0.106 and 1.025 ± 0.106, respectively, with significant differences between groups I and III (p = 0.001). When the mean difference of PSEQ was compared in groups I and III concerning group II, a highly significant p-value was obtained. The mean difference of PSEQ scores in groups I and II concerning group III was -1.025 ± 0.106 and -0.292 ± 0.106, respectively, with a significant p-value, as shown in Table 5.

**Table 5:** Post hoc comparison of PSEQ after intervention. PSEQ: Pain Self-Efficacy Questionnaire.

Groups	After intervention		P-value
		Mean difference (standard error)	
Group I (high-intensity training)	Group II	-30.950 (1.049)	0.001
	Group III	-11.850 (1.049)	0.001
Group II (low-intensity training)	Group I	30.950 (1.049)	0.001
	Group III	19.100 (1.049)	0.006
Group III (control group)	Group I	11.850 (1.049)	0.001
	Group II	-19.100 (1.049)	0.006

## Discussion

TMDs have a high prevalence, affecting quality of life in all age groups. The etiology of these disorders is multidimensional and involves various factors: developmental, environmental, psychological, sleep, lifestyle, etc. To manage pain, TMD patients’ physical therapy has a crucial role [17,18]. To explore the association of physical training with the incidence of TMDs, the present study was carried out among 60 patients with ages ranging from 18 to 40 years. In this study, the prevalence of TMD was higher in females (61.9%) as compared with males (38.1%). When the severity of TMD was assessed by FAI, it was found that 15 (25.0%) patients had mild TMD, 33 (55.0%) had moderate TMD, and 12 (20.0%) had severe TMD; thus, symptoms of moderate TMD were most prevalent in this study. In contrast, several trials have reported that symptoms of mild TMD were most common among their study populations [19-21].

VAS mean score of pain intensity increased among high-intensity training workouts in group I, while in the low-intensity training group, pain was significantly reduced. This showed that on intervention, signs and symptoms of TMDs were aggravated due to high-intensity workouts. Similarly, Chun et al. found that moderate to high-intensity exercises are associated with the worsening of TMD symptoms, while light exercises are favorable [19]. Mansour Ibrahim et al. also proved that vigorous physical training like weight lifting can cause pushing of the lower jaw against the upper jaw, especially in athletes, resulting in myofascial pain and discomfort [20]. The WHO data also support that the lowest amount of physical activity is recommended for wide-ranging health enhancement and in some situations, vigorous activities are harmful [21]. As per Freiwald et al., it has been documented that the occurrence of TMDs is high among athletes as compared to non-athletes [22].

A study on the Korean population by Rhim et al. revealed that low BMI and abdominal obesity are associated with TMD [23]. Eklund also reported that obesity can cause inflammation at low levels, which could be a reason for pain in TMDs [24]. In contrast to these findings, it has been observed that C-reactive protein levels are inversely proportional to the time spent engaging in physical exercises and according to the literature, high physical intensity training is associated with low inflammatory mediators [25,26], although the exact reason for these interactions is not fully understood.

In the etiology of TMD, the sleep cycle also plays an important role. It is encountered that physical activity has a two-way relationship with sleep, and moderate-level physical workout is advisable as a remedy for sleep problems [27]. Guilleminault et al. found that after introducing four weeks of regular exercise, patients suffering from insomnia took less than seven minutes to fall asleep [28]. Thus, to overcome sleep problems in TMD patients, physical workouts are recommended. In this study, the mean value of the PSEQ score was significantly increased in group II (low-intensity training), while in group I (high-intensity training), the mean value of PSEQ was decreased after intervention. It showed that pain self-efficacy while performing various day-to-day activities was increased among patients doing low-intensity physical workouts. Similar results were observed by Atilgan et al., who found that after introducing yoga-based workouts, neck and joint pain was significantly reduced with an increased range of motion in TMD patients [29]. Another study also revealed that face yoga has great efficacy in combating signs and symptoms of TMD [30].

The results of this study showed that low-intensity targeted workouts are very helpful in managing signs and symptoms of TMDs. Literature also supports the idea that coordinated stretching workouts targeting specific muscles can improve jaw functions and lessen the pain of TMD [25-27]. Apart from other factors, lifestyle has an intricate role in the etiology of TMD, as the prevalence of TMD is very high in India, which affects the quality of life. Therefore, inculcating low-intensity physical activities in the form of yoga would be beneficial in managing signs and symptoms of TMD.

Certain limitations of the study need to be acknowledged. The results may not be applicable to larger groups if the selected population is not representative of the overall population. Furthermore, while conclusions can be derived from this research, it is not possible to prove a link between TMDs and the level of physical fitness. The study's conclusions may potentially be impacted by some confounding variables, such as lifestyle choices or genetic susceptibility. Despite these drawbacks, the study maintains higher internal validity because standardized, quantitative measures were used to quantify both exercise intensity and TMD occurrence. It was possible to better understand the dosage-response association between physical training and TMDs by using both high- and low-intensity exercises.

Larger and more diverse populations can be included in future studies to enhance the generalizability of the study findings. Longitudinal studies are advised to evaluate the long-term impact of high- and low-intensity fitness programs on TMDs. A comprehensive understanding of the underlying mechanisms can also be obtained by including psychological evaluations and investigating additional physical activities.

## Conclusions

Based on the results, the present study concluded that signs and symptoms of moderate TMD were prevalent among populations. High-intensity physical training is associated with increased symptom severity; the signs and symptoms of TMD are aggravated by performing vigorous exercises. On the other hand, low-intensity training is helpful to combat painful TMD. In this study, pain intensity was reduced, and self-efficacy was significantly increased while performing various activities among patients in the low-intensity training group. Thus, the present study recommended low-intensity physical workouts with a combination of medications as a remedial measure in TMD. However, the etiology of TMDs is multifactorial; therefore, the evaluation of psychosocial factors, sleep, and lifestyle is also very important to identify a more precise relationship with TMDs.
